# The cost of antiretroviral therapy in Haiti

**DOI:** 10.1186/1478-7547-6-3

**Published:** 2008-02-14

**Authors:** Serena P Koenig, Cynthia Riviere, Paul Leger, Patrice Severe, Sidney Atwood, Daniel W Fitzgerald, Jean W Pape, Bruce R Schackman

**Affiliations:** 1 Division of Social Medicine and Health Inequalities, Brigham and Women's Hospital, Boston, MA 02115, USA; 2 Groupe Haitien d'Etude du Sarcome de Kaposi et des Infections Opportunistes (GHESKIO) Center, Port-Au-Prince, Haiti; 3 Department of Medicine, Weill Medical College, Cornell University, New York, NY, 10021, USA; 4 Department of Public Health, Weill Medical College, Cornell University, New York, NY, 10021, USA

## Abstract

**Background:**

We determined direct medical costs, overhead costs, societal costs, and personnel requirements for the provision of antiretroviral therapy (ART) to patients with AIDS in Haiti.

**Methods:**

We examined data from 218 treatment-naïve adults who were consecutively initiated on ART at the GHESKIO Center in Port-au-Prince, Haiti between December 23, 2003 and May 20, 2004 and calculated costs and personnel requirements for the first year of ART.

**Results:**

The mean total cost of treatment per patient was $US 982 including $US 846 in direct costs, $US 114 for overhead, and $US 22 for societal costs. The direct cost per patient included generic ART medications $US 355, lab tests $US 130, nutrition $US 117, hospitalizations $US 62, pre-ART evaluation $US 58, labor $US 51, non-ART medications $US 39, outside referrals $US 31, and telephone cards for patient retention $US 3. Higher treatment costs were associated with hospitalization, change in ART regimen, TB treatment, and survival for one year. We estimate that 1.5 doctors and 2.5 nurses are required to treat 1000 patients in the first year after initiating ART.

**Conclusion:**

Initial ART treatment in Haiti costs approximately $US 1,000 per patient per year. With generic first-line antiretroviral drugs, only 36% of the cost is for medications. Patients who change regimens are significantly more expensive to treat, highlighting the need for less-expensive second-line drugs. There may be sufficient health care personnel to treat all HIV-infected patients in urban areas of Haiti, but not in rural areas. New models of HIV care are needed for rural areas using assistant medical officers and community health workers.

## Background

Annual expenditures on HIV/AIDS interventions in resource-poor countries have increased 30-fold over the last ten years, to an estimated $US 10 billion in the 2007 [1]. As HIV/AIDS services are rapidly scaled up, determining the cost of specific HIV/AIDS interventions is critical in order to evaluate their cost-effectiveness, set program priorities, and budget for further scale-up [2]. Antiretroviral therapy (ART) is extremely effective in resource-poor settings, increasing survival for patients with AIDS from 30% to 90% at one-year [3-7]. However, the only studies reporting data on the costs of providing ART in resource-poor settings derived from actual patient experience come from South Africa, Thailand, and Mexico [8-11]. Few studies have been conducted in the least developed countries [12-14] and none of these studies collected patient-level data. Cost data are a necessary prerequisite to evaluating the cost-effectiveness of HIV treatment in these severely resource-constrained settings, which account for the greatest share of the global burden of HIV disease.

In the current study, we determine direct medical costs, overhead costs, societal costs, and personnel requirements for providing ART to patients with AIDS in Haiti. Haiti is the poorest country in the Western Hemisphere, on par with the least developed countries in sub-Saharan Africa, with a *per capita *income of $US 380/year and *per capita *health expenditure of $US 8/year [15]. The prevalence of HIV in Haiti is currently estimated at approximately 3.8% in the adult population, about half that of sub-Saharan Africa, which has a regional prevalence of 5.9%, ranging from 3.7% in Angola to 33% in Swaziland [16,17]. In March 2003, the Global Fund to Fight AIDS, Tuberculosis, and Malaria (GFATM) granted funding to Haiti to provide comprehensive HIV/AIDS care which allowed GHESKIO to begin enrolling large numbers of patients on ART. The United States President's Emergency Plan for AIDS Relief (PEPFAR) provided additional support for the project in January 2004. Previous reports from Haiti have documented that ART is effective [4,6]. This report details the cost and personnel requirements of providing ART during the first year of therapy.

## Methods

### Study setting

The Haitian Study Group for Kaposi's Sarcoma and Opportunistic Infections (GHESKIO) was formed in 1982 by a group of Haitian health professionals who described the first cases of AIDS in a developing country [18]. The mission of GHESKIO is to provide clinical services and training, and to conduct research on HIV/AIDS. The clinic is located in downtown Port-au-Prince; the majority of patients are urban residents who make less than US$ 1 per day. The clinic at GHESKIO has provided free HIV counseling, testing, care, and prevention services since 1983. Beginning in March 2003, the clinic began providing ART free-of-charge to all patients with AIDS.

All patients presenting to GHESKIO receive HIV voluntary counseling and testing and screening for other sexually transmitted diseases. All patients with cough undergo evaluation for tuberculosis (TB). HIV-infected patients have a history and physical examination by a physician and a CD4 cell count is measured. Patients with an AIDS-defining illness or a CD4 cell count < 200 cells/ml are started on ART. Prior to starting ART, a complete blood count, renal function tests and liver enzyme tests are performed, and patients meet with a social worker for ART counseling and education.

In the GHESKIO ART treatment protocol, patients on ART are to be seen in the clinic weekly for the first two months of therapy and then monthly thereafter. At each visit, patients are seen by a clinician (physician or nurse), and by a pharmacist who dispenses ART and provides adherence counseling. At three-month intervals, the patients are to be seen by a social worker for additional counseling. Laboratory monitoring includes CD4 cell counts, liver function tests, renal and glucose tests, and complete blood counts every six months. If an opportunistic infection is suspected, appropriate laboratory testing is performed.

Patients are referred to on-site sexually transmitted infection (STI) and family planning clinics when clinically indicated. TB medications are prescribed in the ART clinic and dispensed by a nurse in the adjoining GHESKIO TB clinic. Patients requiring inpatient care are referred to one of several nearby hospitals in Port-au-Prince. When sub-specialty care is required (ophthalmology, dermatology, etc), patients are referred to private providers affiliated with GHESKIO. Patients with wasting or economic need receive nutritional supplementation free-of-charge on site once per month. All patients receive pre-paid telephone cards and transportation subsidies to facilitate retention in the program. Clinic staff give the patients pre-paid telephone cards to enhance adherence to medications and clinic visits; with the cards, the patients can call the clinic staff free-of-charge.

### Health services utilization

We determined actual health care services utilization of consecutively enrolled treatment-naïve adult AIDS patients who initiated ART at the GHESKIO clinic from December 23, 2003 to May 20, 2004. We measured service utilization and cost from the societal perspective, and included all costs that were actually incurred in the month prior to ART initiation, and for 365 days afterwards. We collected data from electronic medical records and from patient charts. For each clinic visit, we extracted: weight; symptoms; active medical conditions; laboratory and radiographic tests conducted; ART medications and number of pills dispensed; medications for TB, opportunistic infections, and other conditions; nutritional support; type of provider seen; referrals to specialty care; and hospitalization.

Cost estimates were obtained using the micro-costing approach outlined by Drummond et al. [19] in which each component of health care that is utilized (such as medications and laboratory tests) is recorded and a unit cost is applied to each component. Costs were measured in 2004 United States dollars. The cost of ART drugs, antibiotics, and other medications stocked in the GHESKIO pharmacy were provided by GHESKIO. For TB and reproductive health medications provided free-of-charge through the Haitian Ministry of Health, we used the prices listed by the International Dispensary Association, a non-profit supplier of generic medications to Haiti. The costs of medications not available at the GHESKIO clinic were obtained from a survey of six private pharmacies in Port-au-Prince. The cost of laboratory tests (including CD4 cell counts) that were conducted on-site were obtained from internal GHESKIO accounting. Chest radiographs were conducted off-site; we used the price that GHESKIO paid for each X-ray. Nutrition costs were provided by the non-governmental organization that provides food supplements to the GHESKIO clinic, and pre-paid telephone card costs were the price paid for them by GHESKIO. For cost of hospitalizations, a panel of GHESKIO clinicians (PL, PS, DWF, and JWP) estimated services utilized on a case-by-case basis; we estimated the amount paid for the day of admission and for subsequent days in care. To estimate the cost of each laboratory test, medication, and radiographic study that was conducted in the hospital, we used the GHESKIO cost to deliver these services. For specialty care, we surveyed specialty physicians in Port-au-Prince to determine the amounts paid for services by these outside providers; we used charges as they were consistent with the labor and administrative costs of comparable visits to GHESKIO physicians.

To determine medical personnel utilization and direct labor costs in the ART clinic, we conducted an observational study of eight physicians and five nurses to estimate the average amount of time per visit. Seventy-seven physician visits and 48 nursing visits were observed by a member of the study team, who recorded the number of minutes each patient spent in the room with each provider. To limit observational bias, measurements were conducted from the waiting room at unannounced dates and times. We found that on average physicians spent 15 minutes and nurses spent 18 minutes with each patient. We interviewed the TB clinic staff to estimate the time spent by the TB nurse to dispense medications and provide adherence counseling (10 minutes per patient). To estimate the indirect labor cost (for patient care activities such as recording patient data), we added an additional 50% to the time devoted to face-to-face medical care (i.e. 30 seconds indirect time for each minute of face-to-face time) based on provider estimates. We then used the GHESKIO average salary by provider type to determine the cost of labor per patient based on a provider work schedule of 200 eight-hour days per year.

Overhead costs were allocated to each visit from actual expenditures based on clinic patient volume (adjusted for the duration of patient visits) except for electricity and cleaning supply costs, which were allocated based on square footage. Capital costs were annualized and discounted at a rate of 5%. These expenditures had been documented in financial reports to the GFATM. Societal costs included non-medical costs (transportation and time) incurred by the patient. The value of patient time was calculated based on the average wage rate per day reported by GHESKIO patients, and transportation costs were calculated according to the patient's residence zone using a weighted average based on the social worker's estimation of the percentage of patients who used each type of transportation (walking, public transportation or private taxi) and its cost (in the case of public transportation or taxi) for patients arriving from each of 16 Port-au-Prince residence zones.

### Analysis

Data were entered into an Access database (Microsoft, Redmond, WA) and then converted to SAS Version 9.1 (SAS, Carey NC) for analysis. The primary outcome was cost of treatment. Direct costs were presented as mean and median costs with interquartile ranges (see Tables 1 and 2). Secondary outcomes included physician time and nursing time spent caring for patients. For both primary and secondary outcomes, we conducted univariate and multivariate analyses using the following variables: CD4 cell count (measured as a continuous variable), number of symptoms at treatment initiation, clinical CDC stage (A, B, C), education (measured as none, primary, secondary, or university), income (in five pre-defined income categories), baseline weight (in quartiles), and age greater than median, baseline hemoglobin <10 mg/dl, mortality, hospitalization, TB treatment, change in regimen, and gender (all measured as binary variables). To examine the impact of early death and loss to follow-up, we repeated these analyses for only the patients who remained in care for the entire year. The arithmetic and geometric means of the total cost and labor (physician time and nurse time) were not normally distributed. Therefore, we conducted the analyses with a generalized linear model (GLM) with a gamma log link [20]. Variables were maintained in the multivariate GLM models for p < 0.05. GLM results are reported as relative differences, representing the ratio of the cost, physician time, or nurse time in the presence of each dichotomized variable (e.g. hospitalization) compared to this value in the absence of each of these variables (e.g. no hospitalization). There were no sets of variables in the reported model that showed signs of unacceptable collinearity.

**Table 1:** Direct treatment utilization and costs for the total cohort (n = 218)

	**Mean utilization per patient for the 1^st ^year of ART**	**Median utilization (IQ range) per patient for the 1^st ^year of ART**	**Mean cost per patient for the 1^st ^year of ART ($US)**	**Median cost (IQ range) per patient for the 1^st ^year of ART ($US)**
Pre-ART work-up (labor and laboratory studies)	*	*	58	61 (61 to 61)
Labor cost (# of ART visits)	12.6	14 (11 to 16)	51	51 (42 to 61)
CD4 cell counts** (# of tests)	1.1	1 (1 to 1)	32	30 (30 to 30)
ART monitoring labs (# of tests)	9.5	11 (6 to 12)	72	81 (46 to 92)
TB tests (# of tests)	1.7	0 (0 to 3)	8	0 (0 to 14)
Other laboratory tests (# of tests)	2.7	2 (1 to 3)	8	5 (0 to 13)
Chest radiographs (# of x-rays)	0.4	(0 to 1)	10	0 (0 to 24)
Anti-retroviral therapy (# of days on ART)	291	362 (241 to 375)	355	282 (225 to 583)
TB medications (# of days on therapy)	19	0 (0 to 0)	6	0 (0 to 0)
Other medications***	*	*	33	28 (15 to 40)
Outside referrals	0.3	0 (0 to 0)	31	0 (0 to 0)
Hospitalizations	0.1	0 (0 to 0)	62	0 (0 to 0)
Telephone cards	2.4	2.4 (1 to 3)	3	3 (1 to 4)
Nutritional support	*	*	117	140 (0 to 180)

**Total (# of days in care)**	**299**	**365 (357 to 365)**	**846**	**846 (654 to 1075)**

IQ range = interquartile range

* No utilization units defined.

** This does not include the baseline CD4 cell count done before the initiation of ART; also, many patients had a 12-month CD4 cell count that was done more than 365 days after ART initiation, and was therefore excluded from the analysis.

*** GHESKIO paid a mean cost of $US 33.69 per patient for other medications (i.e. not including ART or TB drugs). The mean cost per patient for medications purchased outside of GHESKIO was $US 4.31 (median cost of $US 1.23).

**Table 2:** Direct treatment utilization and costs for patients that remained in care for one full year (n = 169)

	**Mean utilization per patient for the 1^st ^year of ART**	**Median utilization (IQ range) per patient for the 1^st ^year of ART**	**Mean cost per patient for the 1^st ^year of ART ($US)**	**Median cost (IQ range) per patient for the 1^st ^year of ART ($US)**
Pre-ART workup (labor and laboratory studies)	*	*	58	61 (61 to 61)
Labor cost (# of ART visits)	14.9	15 (14 to 16)	59	54 (47 to 63)
CD4 cell counts** (# of tests)	1.3	1 (1 to 2)	39	30 (30 to 60)
ART monitoring labs (# of tests)	11.3	11 (9 to 13)	85	85 (68 to 99)
TB tests (# of tests)	1.8	0 (0 to 3)	8	0 (0 to 14)
Other laboratory tests (# of tests)	3.2	2 (1 to 4)	9	8 (2 to 13)
Chest radiographs (# of x-rays)	0.4	0 (0 to 1)	10	0 (0 to 24)
Anti-retroviral therapy (# of days on ART)	357	367 (355 to 379)	439	413 (273 to 601)
TB medications (# of days on therapy)	21	0 (0 to 0)	6	0 (0 to 0)
Other medications***	*	*	37	31 (21 to 42)
Outside referrals	0.4	0 (0 to 0)	39	0 (0 to 19)
Hospitalizations	0.05	0 (0 to 0)	35	0 (0 to 0)
Telephone cards	2.4	3.2 (2 to 3)	3	4 (3 to 4)
Nutritional support	*	*	145	160 (100 to 200)

**Total (# of days in care)**	**364**	**365 (365 to 365)**	**972**	**923 (770 to 1098)**

IQ range = interquartile range

* No utilization units defined.

** This does not include the baseline CD4 cell count done before the initiation of ART; also, many patients had a 12-month CD4 cell count that was done more than 365 days after ART initiation, and was therefore excluded from the analysis.

*** GHESKIO paid a mean cost of US$33.69 per patient for other medications (i.e. not including ART or TB drugs). The mean cost per patient for medications purchased outside of GHESKIO was $US 4.31 (median cost of $US 1.23).

## Results

### Study population

Between December 23, 2003 and May 20, 2004, 220 treatment-naïve adult AIDS patients initiated ART at the GHESKIO clinic. We reviewed the medical records of 218 (99%) of these patients; we were not able to find two records. The baseline characteristics of the 218 study patients are provided in Table 3. Of the 218 patients initiated on ART, 169 (78%) remained in care for one year, 35 (16%) died after a mean time in care of 68 days, and 14 (6%) were lost to follow-up after a mean time in care of 98 days. A total of 20 patients (9%) were hospitalized during the study period, and 27 (12%) had a change in ART regimen due to side effects or treatment failure.

**Table 3:** Baseline characteristics of patients receiving ART (n = 218)

Female sex – no. (%)	122 (56)
Age – no. (%)	
1. <20 yr	2 (2)
2. 20 – 29 yr	25 (12)
3. 30 – 39 yr	83 (38)
4. 40 – 49 yr	74 (34)
5. >49 yr	31 (14)

Resident of Port-au-Prince – no. (%)	191 (88)

Income < $US 1/day – no. (%)	148 (68)

Education – no. (%)	
1. None	49 (22)
2. Primary school	67 (31)
3. Secondary school	94 (43)
4. College	8 (4)

Marital status – (%)	
1. Common-law marriage	65 (30)
2. Married	37 (17)
3. Separated	54 (25)
4. Single	31 (17)
5. Widowed	26 (12)

AIDS-defining illness (CDC Stage C) – no. (%)	141 (65)

Body weight – kg	
1. Men	
a) Median	55.5
b) Interquartile range	49.1 to 61.4
2. Women	
a) Median	48.4
b) Interquartile range	42.7 to 55.5

CD4 cell count/mm^3^	
1. Median	108
2. Interquartile range	40 to 180

CD4 cell count <50/mm^3 ^– no. (%)	61 (30)

Initial antiretroviral-therapy regimen – no. (%)	
1. Nevirapine, zidovudine, lamivudine	111 (51)
2. Other nevirapine-based regimen	5 (2)
3. Efavirenz, zidovudine, lamivudine	92 (42)
4. Other efavirenz-based regimen	10 (5)

### Cost of ART treatment

The mean total cost of treatment per patient was $US 982, including $US 846 in direct costs, $US 114 for overhead, and $US 22 for societal costs. For patients who remained in care for one year, the mean total cost of treatment per patient was $US 1132. Table 1 presents the mean, median, and interquartile ranges of direct costs for all patients in the cohort; Table 2 presents these values for patients who remained in care for one year.

The mean direct cost per patient included: $US 355 for ART; $US 130 for laboratory testing and radiographs; $US 117 for food supplementation; $US 62 for hospitalization; $US 58 for screening evaluation before ART was initiated; $US 51 for labor; $US 39 for non-ART medications; $US 31 for referrals to specialty care outside of the GHESKIO clinic; and $US 3 for telephone cards (Table 1). For patients who remained in care for one year, the mean direct cost was $US 972 (Table 2).

The mean overhead costs were $US 114 per patient; the largest components (Table 4) were indirect clinical costs ($US 26), non-direct health care professional labor ($US 24), monitoring and evaluation ($US 19), electricity ($US 15), and maintenance ($US 15). Mean societal costs were $US 22 per patient. For patients who remained in care for one year, mean overhead costs were $US 135 and mean societal costs were $US 27.

**Table 4:** Indirect ART clinic costs, overhead, and societal costs ($US)

	**Mean cost per patient for total cohort for 1^st ^year ART ($US)**	**Mean cost per patient in care for one year ($US)**
**Indirect ART clinic-related costs**		
Transportation and storage of medications, equipment, and supplies	8	9
ART clinic computers and furniture	18	21
**Overhead costs**		
Monitoring and evaluation	19	22
Non-direct patient care time for health care professionals	24	28
Labor for administrative staff	3	4
Electricity	15	18
Maintenance	15	18
Phone and communication	3	4
Office supplies	5	6
Cleaning supplies	3	4
Security	1	1

Total indirect and overhead costs	**114**	**135**

**Societal costs**		
Transportation to and from GHESKIO*	16	19
Patient Time at GHESKIO	5	6
Societal costs for outside referrals (transportation and patient waiting time)	0.5	1
Societal costs for hospitalization (transportation and waiting time for patients and family members, and cost of death in the hospital)	0.5	1

**Total societal costs**	**22**	**27**

*GHESKIO subsidized transportation fees for all patients ($US 0.60 per ART visit; mean subsidy per patient $US 7.56 per year).

### Predictors of cost: Full cohort

In univariate analyses, predictors of higher treatment cost included hospitalization ($US 1406 vs. $US 939), change in ART regimen due to side effects or treatment failure ($US 1308 vs. $US 936), fewer than five symptoms at ART initiation ($US 1044 vs. $US 853), higher body weight at baseline ($US 1031 vs. $US 825), and survival for one year (Table 5). Patients with high body weight and few symptoms at ART initiation were more likely to survive and remain in care for a full year. Total costs were significantly higher for patients who remained in care ($US 1132) compared with those who died ($US 508) or were lost to follow-up ($US 382), due to additional days of treatment. Cost per day in care was significantly higher for patients who died; they had a higher rate of hospitalization (29% vs. 6%) and their cost per day in care was approximately twice as high ($7.47 per day) as those who remained in care for one year ($3.10 per day) or were lost to follow-up ($3.90 per day).

**Table 5:** Univariate models of cost and medical personnel utilization per patient in the total cohort (n = 218)

**Treatment cost**	**Relative difference**	**95% CI**	**p-value**
Survived during observation period (n = 183)	2.11	(1.71, 2.61)	<0.0001
Hospitalized during ART (n = 20)	1.50	(1.13, 2.01)	0.0052
ART changed due to side effects (n = 27)	1.40	(1.09, 1.79)	0.0083
Weight above lowest quartile at baseline (n = 155)	1.25	(1.03, 1.52)	0.0244
Fewer than 5 symptoms at ART initiation (n = 147)	1.22	(1.03, 1.46)	0.0238
**Physician time**	**Relative difference**	**95% CI**	**p-value**
ART changed due to side effects or failure (n = 27)	1.62	(1.34, 1.97)	<0.0001
Survived during observation period (n = 183)	1.59	(2.18, 3.78)	<0.0001
Treated for TB (n = 33)	1.58	(1.32, 1.88)	<0.0001
Female gender (n = 122)	1.22	(1.07, 1.39)	0.0040
No Income (n = 125)	1.20	(1.05, 1.37)	0.0089

**Nurse time**	**Relative difference**	**95% CI**	**p-value**
Treated for TB (n = 33)	1.56	(1.27, 1.90)	<0.0001
Survived during observation period (n = 183)	2.13	(1.68, 2.70)	<0.0001

In multivariate analyses, hospitalization, a change in ART regimen, TB treatment, and survival for one year were significantly predictive of higher treatment costs (Table 6). Survival was associated with higher total treatment costs because these patients remained in care and used services for an entire year.

**Table 6:** Multivariate models of cost and medical personnel utilization per patient in the total cohort (n = 218)

**Treatment cost**	**Relative difference**	**95% CI**	**p-value**
Survived during observation period (n = 183)	3.37	(2.72, 4.17)	<0.0001
Hospitalized during ART (n = 20)	2.49	(1.90, 3.25)	<0.0001
Treated for TB (n = 33)	1.26	(1.03, 1.54)	0.0275
ART changed due to side effects (n = 27)	1.25	(1.00, 1.56)	0.0491
**Physician time**	**Relative difference**	**95% CI**	**p-value**
Survived during observation period (n = 183)	1.97	(1.33, 2.69)	<0.0001
Treated for TB (n = 33)	1.54	(1.32, 1.88)	<0.0001
ART changed due to side effects or failure (n = 27)	1.30	(0.68, 1.97)	0.0127
Hospitalized during ART (n = 20)	1.27	(1.01, 1.62)	0.0492
Female Gender (n = 122)	1.21	(1.12, 1.50)	0.0005

**Nurse time**	**Relative difference**	**95% CI**	**p-value**
Survived during observation period (n = 183)	2.13	(1.68, 2.70)	<0.0001
Treated for TB (n = 33)	1.56	(1.27, 1.90)	<0.0001

### Predictors of cost: Patients who remained in care for one year

In univariate analyses of patients who remained in care for one year, higher costs were associated with hospitalization ($US 1955 vs. $US 1092), a change in ART regimen due to side effects ($US 1469 vs. $US 1085), TB treatment ($US 1336 vs. $US 1105), and CDC Stage C AIDS symptoms at ART initiation ($US 1194 vs. $US 1048) (Table 7). In multivariate analysis, hospitalization, change in ART regimen, and older age were associated with significantly higher treatment costs (Table 8).

**Table 7:** Univariate models of cost and medical personnel utilization per patient that remained in care for one full year (n = 169)

**Treatment cost**	**Relative difference**	**95% CI**	**p-value**
Hospitalized during ART (n = 8)	1.79	(1.40, 2.28)	<0.0001
ART changed due to side effects (n = 21)	1.35	(1.15, 1.59)	0.0002
Treated for TB (n = 20)	1.2	(1.02, 1.43)	0.0276
CDC Stage C (n = 98)	1.14	(1.02, 1.27)	0.0199
**Physician time**	**Relative difference**	**95% CI**	**p-value**
Treated for TB (n = 20)	1.83	(1.52, 2.21)	<0.0001
ART changed due to side effects (n = 21)	1.36	(1.01, 1.87)	<0.0001
CDC Stage C (n = 98)	1.35	(1.18, 1.54)	<0.0001
No income (n = 94)	1.21	(1.06, 1.39)	0.0046
Hemoglobin <10 mg/dl at baseline (n = 44)	1.18	(1.01, 1.38)	0.0321
Body weight in the bottom quartile at baseline (n = 33)	1.19	(1.01, 1.41)	0.0479
Female gender (n = 94)	1.16	(1.01, 1.33)	0.0301

**Nurse time**	**Relative difference**	**95% CI**	**p-value**
Treated for TB (n = 20)	2.00	(1.70, 2.33)	<0.0001
ART changed due to side effects (n = 21)	1.21	(1.01, 1.46)	0.0437
CDC Stage C (n = 98)	1.18	(1.04, 1.33)	0.0309

**Table 8:** Multivariate models of cost and medical personnel utilization per patient that remained in care for one full year (n = 169)

**Treatment cost**	**Relative difference**	**95% CI**	**p-value**
Hospitalized during ART (n = 8)	1.71	(1.34, 2.19)	<0.0001
ART changed due to side effects (n = 21)	1.22	(1.03, 1.45)	0.0176
Age greater than median (n = 84)	1.14	(1.03, 1.27)	0.0126
**Physician time**	**Relative difference**	**95% CI**	**p-value**
Treated for TB (n = 20)	1.60	(1.28, 2.00)	<0.0001
ART changed due to side effects or failure (n = 21)	1.25	(1.01, 1.57)	0.0500
Hemoglobin < 10 mg/dl (n = 44)	1.22	(1.04, 1.43)	0.0139

**Nurse time**	**Relative difference**	**95% CI**	**p-value**
Treated for TB (n = 20)	1.91	(1.61, 2.26)	<0.0001

### Medical personnel utilization

The mean total physician labor time per patient was 2.4 hours, and total nurse labor time per patient was 4.0 hours, including the time of the TB clinic nurse. Assuming 1600 hours per year available for patient care by each doctor or nurse, this implies that 1.5 doctors and 2.5 nurses are required to treat 1000 patients in the first year after initiating ART.

In univariate analyses of the total cohort, both physicians and nurses spent more time with patients with TB, and with those who survived (Table 5). Physicians also devoted significantly more time to patients with a change in ART regimen, no income, and female gender. Patients who died consumed more physician time per day in care (1.2 minutes per day in care) than those who were lost to follow up (0.8 minutes per day in care) or those who remained in treatment for the first year (0.5 minutes per day in care). For nurses, there was little difference in time per day in care among these patient groups. Nurses spent 1.4 minutes per day in care for patients who died, 1.2 minutes for those who were lost to follow-up, and 1.3 minutes for those who remained in care for the year.

In the multivariate analysis of the total cohort, survival and TB treatment were associated with greater physician and nursing time, and a change in ART regimen, hospitalization, and female gender were associated with more physician time (Table 6). In the multivariate analysis of those who remained in care for the whole year, TB treatment was associated with increased physician and nursing time, and patients who changed ART regimen and those who were anemic required more physician time (Table 8).

## Discussion

We determined health care utilization, cost, and human resource requirements for the first year of ART in a cohort of adult AIDS patients consecutively enrolled in an urban clinic in Port-au-Prince, Haiti. HIV/AIDS can be effectively treated in Haiti for approximately US$1000 per patient per year.

The cost of ART medications comprised only 36% of the total expenditures and is not the only driver of cost. In Haiti, patients received generic medications at an average cost of $US 1.22 per day for a three-drug antiretroviral regimen. This is similar to a recent study from South Africa, which reported ART costs of $US 1.25 per day, with ART consuming 23% of the budget [10]. These findings are in stark contrast to published figures from countries where generic antiretroviral medications are not routinely used [21]. In a study conducted in the United States, ART medications accounted for 73% of lifetime medical costs [22]. In a study conducted in Mexico, ART brand named drugs comprised 85% of the total treatment cost for the first year on therapy [9].

A change in ART regimen due to side effects or treatment failure was associated with significantly higher treatment costs due to the high cost of providing a second-line regimen. Many second-line drugs are not available in generic formulations. For example, the second-line ART drugs lopinavir/ritonavir and abacavir cost $US 2 and $US 3 per day each. Furthermore, medication changes required additional physician time. This highlights the need for less-toxic first-line regimens and less-expensive generic second-line drugs for resource-poor settings.

Laboratory tests for the early detection of drug toxicities and for monitoring response to therapy consumed approximately 15% of the total cost; however, the utility of these lab tests is not certain. A recent study conducted in Thailand investigated the necessity for laboratory toxicity monitoring of ART and concluded that just two lab tests, an ALT and hemoglobin, could prevent most serious short-term toxicity [23]. If this study is confirmed, then laboratory monitoring of toxicities could be simplified and resources saved. Monitoring for treatment failure is necessary to prevent individual patient morbidity and mortality, and to prevent emergence of drug-resistant virus strains. However, a study conducted in Canada suggests that CD4 cell criteria are neither sensitive nor specific in predicting which patients have failed to achieve virologic suppression [24]. In contrast, a study conducted in Africa suggests that inexpensive ELISA based p-24 antigen assays may be very predictive of virologic failure [25]. Using such a simple and inexpensive test to detect virologic failure could improve the quality of HIV care and reduce cost compared with current recommendations to monitor CD4 cell counts every six months, if these findings are confirmed [26].

Nutritional support accounted for approximately 10% of the total cost. This expenditure is critical because Haiti suffers from one of the highest rates of food insecurity in the world, with nearly two-thirds of its population undernourished [15,27]. Wasting is the most common cause of death in patients with AIDS in Haiti [6]. Studies suggest that poor nutrition can accelerate HIV disease progression and death, and that nutritional support may be an effective intervention for malnourished HIV-infected patients [28]. Further, we have found that provision of nutritional support improves adherence to ART and retention in ART treatment [4]. Transportation subsidies are also critical. On average, GHESKIO pays half of the transportation cost, and subsidizes the full amount for patients who miss clinic visits for economic reasons.

In Haiti, we found that the cost of care was significantly higher for patients who survived an entire year ($US 1132) than for patients who died (US$ 508). This was due to the additional number of days in care, even though those who died had a higher rate of hospitalization, and their cost per day in care was over twice as high as those who remained in care for the full year. Most patients who died on ART did so soon after ART initiation, and access to advanced tertiary medical care was unfortunately extremely limited; the situation is similar in other least developed countries worldwide. This finding differs from results from better-resourced settings, where hospitalization, advanced diagnostic tests, intensive care, and medications for extremely ill patients result in high costs during the last months of life [9,10,29,30]. Patients in this study had relatively advanced HIV/AIDS, with a median CD4 cell count of 108 at study entry; costs may differ in other settings where patients initiate care with less advanced disease [21,29,30].

The model of ART employed at GHESKIO requires 1.5 physicians and 2.5 nurses to care for 1000 patients. Haiti has approximately 2,000 doctors and most of them are concentrated in urban areas. Therefore, the GHESKIO model may be feasible in the capital city of Port-au-Prince and other urban centers. For example, it is estimated that there are approximately 60,000 HIV-infected people in Port-au-Prince. If half of them required ART, then 45 physicians and 75 nurses would be needed to provide care. However, in rural areas, where there are far fewer physicians and nurses, the GHESKIO model may not be feasible. Models of ART using assistant medical officers, community health care workers, and a limited number of nurses and physicians are needed.

Integration of HIV and TB treatment programs may be a way to conserve limited resources and health professional time. In our cohort, co-treatment of tuberculosis and HIV required only a small increase in cost and physician time compared with treating HIV alone. Others have argued that HIV and TB programs in Africa and other resource-poor settings have many synergies, and that integration may make both programs more effective [31,32]. Reproductive health services can also be integrated into the HIV/AIDS clinic. We found that female gender was associated with increased physician time because women were more likely to receive reproductive health services, including treatment in the adjoining sexually transmitted infections clinic.

Our study was limited to one clinic in Port-au-Prince. Treatment costs and human resource demands will likely vary from clinic to clinic. Therefore, alternative models of care, especially in rural areas, should be the subjects of future cost and outcomes studies. The costing methodology was limited because we used charges instead of costs for radiographs and specialty care performed outside of GHESKIO, clinician estimates of hospital service utilization, and clinic costs as a proxy for the cost of ancillary services provided in the hospital. However, these limitations are unlikely to have significantly biased our estimates because of the small proportion of total costs represented by hospitalizations and other medical services performed outside of the GHESKIO clinic.

In summary, HIV/AIDS can be treated in Haiti in the first year after initiation of ART for about $US 1000 per patient. With generic first-line antiretroviral drugs, only 36% of the cost was for medications. Patients who change regimens are significantly more expensive to treat, highlighting the need for less expensive second-line drugs. This model of ART delivery requires 1.5 physicians and 2.5 nurses to care for 1000 patients. There may be sufficient health care personnel to treat all HIV-infected patients using this model in urban areas of Haiti, but not in rural areas. New models of HIV care for rural areas using assistant medical officers and community health workers are needed. The implications of these findings go beyond Haiti, to the other least developed countries worldwide, where tertiary care is frequently unavailable, health care personnel are scarce in rural areas, and there is a high prevalence of TB. The total resources required to provide care will be considerably higher in high HIV/AIDS prevalence countries of sub-Saharan Africa than in Haiti, and the shortage of qualified health care personnel in these countries will place an even greater burden on the ability to deliver adequate care.
